# Prognostic Value of HFA-PEFF Score in Patients Undergoing Transcatheter Aortic Valve Implantation

**DOI:** 10.7759/cureus.27152

**Published:** 2022-07-22

**Authors:** Sultan Alotaibi, Karim Elbasha, Martin Landt, Jatinderjit Kaur, Arief Kurniadi, Mohamed Abdel-Wahab, Ralph Toelg, Gert Richardt, Abdelhakim Allali

**Affiliations:** 1 Heart Center, Segeberger Kliniken, Bad Segeberg, DEU; 2 Cardiac Center, King Fahad Armed Forces Hospital, Jeddah, SAU; 3 Health Policy, London School of Economics and Political Science (LSE), London, GBR; 4 Department of Structural Heart Disease/Cardiology, Heart Center Leipzig, Leipzig, DEU

**Keywords:** tavr', heart failure, aortic stenosis, hfa-peff, hfpef, tavi

## Abstract

Background

The HFA-PEFF score may help in predicting long-term outcomes in patients undergoing transcatheter aortic valve implantation (TAVI) for severe aortic stenosis and preserved left ventricular ejection fraction (EF).

Methods

We retrieved data from 1,332 patients undergoing TAVI between 2010 and 2019 from the Prospective Segeberg TAVI Registry (ClinicalTrials.gov Identifier: NCT03192774). We calculated the HFA-PEFF score for 1,022 patients who had preserved EF (≥50%). To assess the prognostic value of the HFA-PEFF score in predicting adverse events, we dichotomised the patients according to a cut-off score of five (score <5 group: n=528 (51.6%), score ≥5 group: n=494 (48.3%)).

Results

The HFA-PEFF score ≥5 groups were older (81.9±6.3 years vs. 80.3±6.9 years; p<0.001) and had a higher prevalence of atrial fibrillation (35.1% vs 20.8%; p<0.001) and chronic kidney disease (30.1% vs 26.1%; p<0.001). Kaplan-Meier survival analyses over 24 months showed increased cardiovascular (CV) mortality (12.5% vs. 7.7%, log-rank; p=0.028) and first heart failure-related rehospitalisation (7.7% vs. 4.0%, log-rank p=0.014) in the HFA-PEFF score ≥5 groups compared with those of lower scores. No significant difference in all-cause mortality between both groups was observed (22.0% vs. 17.9%, log-rank p=0.127). In multivariate analysis, HFA-PEFF score ≥5 failed to predict CV mortality (aHR 1.37, 95% CI: 0.90-2.08, p=0.140) and time to first heart failure-related rehospitalisation (aHR 1.49, 95% CI: 0.83-2.65, p=0.181).

Conclusion

The HFA-PEFF score showed limited value in predicting long-term mortality and adverse heart failure-related events in patients with preserved EF undergoing TAVI. Clinical variables specific to this population could complement the HFA-PEFF score for better risk prediction.

## Introduction

Heart failure with preserved ejection fraction (HFpEF) is a heterogeneous syndrome that occurs in elderly individuals suffering from diverse comorbidities such as valvular heart disease. As opposed to patients with reduced ejection fraction heart failure, identifying patients with HFpEF and predicting their outcome is challenging [1].

HFpEF patients and those with advanced aortic valve stenosis have a broad overlap in their clinical presentation and pathophysiologic background. The left ventricular pressure overload, a similar phenomenon in HFpEF, in patients with severe aortic stenosis eventually leads to structural remodelling and damaging changes in the heart valves and chambers [2]. These functional and structural changes in patients with advanced stages of aortic stenosis can mimic the signs of HFpEF.

Further understanding of HFpEF pathologic processes, coupled with advancements in diagnostic tools, led to the development of comprehensive approaches to help identify patients with suspected HFpEF [1]. Diagnostic criteria were suggested, and some evolved into a stepwise diagnostic approach such as the HFA-PEFF diagnostic algorithm that was endorsed by the Heart Failure Association of the European Society of Cardiology [3]. The HFA-PEFF score proved its generalisability in diagnosing HFpEF patients in two prospective cohorts [4]. Its clinical usefulness was also suggested as a prognostic tool in predicting adverse outcomes in HFpEF patients [5]. Although patients with severe aortic stenosis were excluded in the formulation and validation of the HFA-PEFF score, its prognostic value was recently tested in a single-center cohort study over a period of one year [6].

As transcatheter aortic valve implantation (TAVI) can potentially reverse cardiac remodeling, leading to better outcomes, the concomitant HFpEF components in those patients might hinder this progress in the long term [7]. Our aim was to determine whether the HFA-PEFF score can detect patients who might still suffer from late adverse outcomes related to HFpEF despite being already treated for severe aortic stenosis. We evaluated the long-term prognostic impact of the HFA-PEFF score in patients undergoing TAVI for severe aortic stenosis and preserved left ventricular ejection fraction.

## Materials and methods

Data collection and study design

We retrieved data from our prospective single-centre registry, the Prospective Segeberg TAVI Registry (ClinicalTrials.gov identifier: NCT03192774). Additional data were obtained from in-hospital records, routine follow-up visits, and from the referring physician. The institutional database was approved by the local ethics committee, and informed consent was obtained from all patients. The study was conducted in accordance with principles of good clinical practise and all procedures followed were in accordance with the ethical standards of the Declaration of Helsinki of 1964, as revised in 2013 [8].

We used the proposed points system in the second step of the HFA-PEFF diagnostic algorithm to dichotomise patients into two groups based on a cut-off score of 5. We assumed missing data from any domain as zero in the calculation of the HFA-PEFF score. We decided to exclude the patient if all three domains were missing. The further proposed steps in the algorithm were not analysed in this study.

Patients

Between January 2010 and December 2019, all patients undergoing TAVI for severe aortic stenosis from the Prospective Segeberg TAVI Registry at the Heart Centre Segeberger Kliniken, Germany were examined. The indication for the TAVI procedure was in accordance with the local interdisciplinary heart valve team. Of those, patients who had a preserved left ventricular ejection fraction of ≥50% and fulfilled the pre-test assessment suggested by the first step of the HFA-PEFF algorithm were deemed eligible for inclusion in our study. We calculated the HFA-PEFF score and assigned them to our pre-defined two groups for analysis.

Study endpoints

We examined the prognostic value of a high HFA-PEFF score by comparing all-cause mortality, cardiovascular (CV) mortality, and time to first heart failure-related rehospitalisation between the two groups within 24 months after TAVI. All clinical endpoints were defined according to Valve Academic Research Consortium 3 (VARC-3) criteria [9].

HFA-PEFF Score

The Heart Failure Association of the European Society of Cardiology produced an updated consensus recommendation for diagnosing HFpEF-the HFA-PEFF diagnostic algorithm (heart failure association pre-test assessment, echocardiography and natriuretic peptide, functional testing, and final etiology).

It is an algorithm based on a stepwise approach, which begins with establishing the pre-test likelihood of HFpEF by examining risk factors and assessing exercise intolerance. The point-based approach in the second step of this algorithm incorporates three domains-functional, morphological, and biomarker-to estimate the likelihood of patients suffering from HFpEF. A high-likelihood score (≥5 points) is considered diagnostic for HFpEF, and a low-likelihood score (0 or 1 point) rules out HFpEF. For patients with an intermediate score (2-4), further steps for functional and aetiology evaluation are advised when appropriate [3].

Statistical analysis

We summarised qualitative variables as frequencies and percentages, and quantitative variables as mean ± SD or median (25-75th quartiles), depending on the variable distribution. For continuous data, Student’s t-test or non-parametric tests (Mann-Whitney U) were used to compare characteristics between the two groups according to data distribution, while chi-square or Fischer’s exact tests were used for comparison of qualitative data. Survival curves were produced using the Kaplan-Meier method, and the difference between the hazards was tested using the log-rank test. Univariate analysis was done using Cox regression analysis, and the hazard ratio (HR) and the 95% confidence interval (CI) were presented. Multivariate Cox regression analysis was performed using entry criteria of p < 0.1 in univariate analysis. For the univariate analyses, the following set of variables was used: age, sex, body mass index, diabetes mellitus, hypertension, hyperlipidaemia, chronic kidney disease, glomerular filtration rate, atrial fibrillation, coronary artery disease, previous myocardial infarction, previous percutaneous coronary intervention, previous coronary artery bypass graft, complete coronary revascularisation before TAVI, presence of peripheral artery disease, previous stroke, chronic obstructive pulmonary disease (COPD), New York Heart Association (NYHA) stage, surgical risk scores (EuroSCORE II and Society of Thoracic Surgeons (STS) PROM score), mean aortic valve pressure, moderate to severe mitral regurgitation, and procedural access. Statistical analysis was performed using SPSS v. 24 software (IBM, Armonk, NY, U.S.A).

## Results

Characteristics of the study cohort

**Figure 1:**
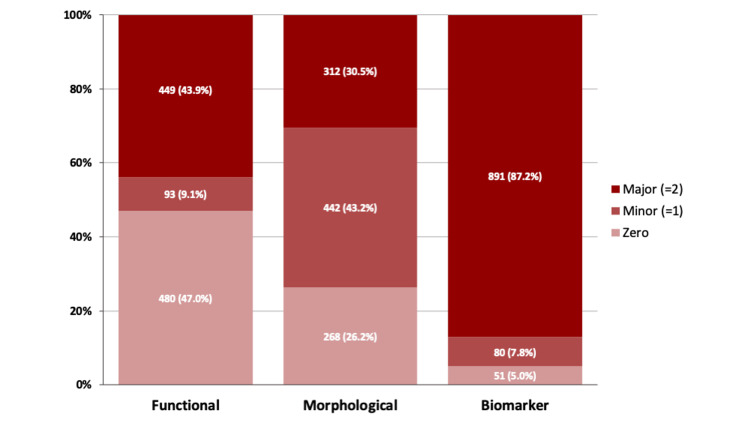
Distribution of the individual domains of the HFA-PEFF score Bar graphs show the distribution of the individual domains (functional, morphological, and biomarker) of HFA-PEFF score in the study population of the Prospective Segeberg TAVI Registry. Data are expressed as numbers (percentage). Major criteria= 2 points, minor criteria= 1 point, zero as no points.

Our study cohort contained 1,332 consecutive patients who underwent TAVI. Of those, 1,022 patients (76.7%) had a preserved left ventricular ejection fraction of ≥50% and were included in the analysis. Around half of the patients had higher HFA-PEFF scores (total score ≥5 group: n=494 (48.3%); total score <5 group: n=528 (51.6%)). The distribution of the three domains of the HFA-PEFF score among the study population is shown in Figure 1. There was no missing data that prevented the score from being calculated. Patients in the HFA-PEFF score ≥5 groups were older (81.9±6.3 years vs. 80.3±6.9 years; p<0.001) and had a higher prevalence of both atrial fibrillation (35.1% vs 20.8%; p<0.001) and chronic kidney disease, defined as kidney damage or glomerular filtration rate <60 mL/min/1.73 m^2^ (30.1% vs 26.1%; p<0.001). Patients in the HFA-PEFF score ≥5 groups presented with advanced NYHA stages and had higher estimated surgical risk. Baseline characteristics of the study cohort are presented in Table 1.

**Table 1:** Baseline characteristics BMI: body mass index; DM: diabetes mellitus; CKD: chronic kidney disease; GFR: glomerular filtration rate; CKD-EPI: Chronic Kidney Disease-Epidemiology Collaboration; CAD: coronary artery disease; MI: myocardial infarction; PCI: percutaneous coronary intervention; CABG: coronary artery bypass graft; PAD: peripheral artery disease; COPD: chronic obstructive pulmonary disease; NYHA: New York Heart Association; STS PROM: Society of Thoracic Surgeons Predicted Risk of Mortality; MR: mitral regurgitation; TF: transfemoral.

	All patients n=1022	HFA-PEFF score ≥5 n=494(48.3%)	HFA-PEFF score <5 n=528(51.6%)	P-value
Clinical				
Age (years)	81.09 ±6.3	81.9±5.6	80.3±6.9	<0.001
Female sex (%)	575 (56.3)	298 (60.3)	277 (52.4)	0.012
BMI (kg/m^2^)	26.8 [24.1–30.4]	26.5 [23.6–29.5]	27.1 [24.3–31.1]	0.023
DM (%)	262 (25.6)	130 (26.3)	132 (25.0)	0.667
Hypertension (%)	908 (88.8)	445 (90.0)	463 (87.6)	0.234
Hyperlipidaemia (%)	432 (42.3)	200 (40.4)	232 (43.9)	0.282
CKD (%)	575 (56.3)	308 (62.3)	267 (50.5)	<0.001
GFR (CKD-EPI)	56.8 [43.6–73.4]	54.1 [40.5–68.6]	59.4 [46.3–77.1]	<0.001
Atrial fibrillation (%)	359 (35.1)	213 (43.1)	146 (27.6)	<0.001
CAD (%)	631 (61.7)	301 (60.9)	330 (62.5)	0.607
1VD (%)	209 (20.5)	102 (33.8)	107 (32.4)	
2VD (%)	198 (19.4)	88 (29.2)	110 (33.3)	
3VD (%)	216 (21.1)	108 (35.8)	108 (32.7)	0.845
Previous MI	74 (7.2)	39 (7.89)	35 (6.6)	0.465
Previous PCI	336 (32.9)	165 (33.4)	171 (32.3)	0.739
Previous CABG	141 (13.8)	64 (12.9)	77 (14.5)	0.469
Complete revascularisation	364 (35.6)	168 (34.0)	196 (37.1)	0.284
PAD	165 (16.1)	81 (16.3)	84 (15.9)	0.865
Previous stroke	107 (10.5)	51 (10.3)	56 (10.6)	0.919
COPD	126 (12.3)	67 (13.5)	59 (11.1)	0.255
NYHA III	456 (44.6)	249 (50.4)	207 (39.2)	<0.001
NYHA IV	60 (5.9)	32 (6.4)	28 (5.3)	<0.001
Euro SCORE II	3.34 [1.9–5.8]	3.9 [2.4–6.7]	2.7 [1.7–5.1]	<0.001
STS PROM	3.5 [2.4–5.3]	4.1 [2.8–6.1]	2.9 [2.1–4.6]	<0.001
Echocardiographic				
Aortic valve Pmean (mmHg)	44.2±16.1	45.1±17.3	43.2±14.9	0.064
Moderate or severe MR	54 (5.3)	33 (6.6)	21 (3.9)	0.068
Procedural access				
TF access	1007 (98.5)	487 (98.5)	520 (94.4)	0.839
Other access	15 (1.5)	7 (1.4)	8 (1.5)	

Outcomes according to HFA-PEFF score

**Figure 2:**
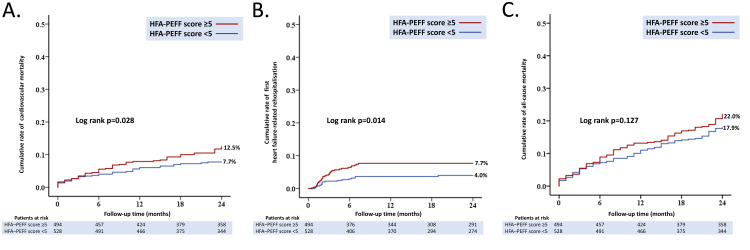
Adverse events after TAVI according to HFA-PEFF score Kaplan–Meier curves showing the cumulative rate over 24 months of (A) cardiovascular mortality; (B) first heart failure-related rehospitalisation; (C) all-cause mortality stratified by HFA-PEFF score (≥5 versus <5) from the study population of the Prospective Segeberg TAVI Registry.

The cumulative CV mortality at two years was higher in the HFA-PEFF score ≥5 group compared with the HFA-PEFF score <5 groups (12.5% vs. 7.7%, HR 1.58, 95% CI: 1.05-2.38, p=0.028) (Figure 2A). Also at two years, the cumulative risk of first heart failure-related rehospitalisation was higher in the HFA-PEFF score ≥5 group compared with the lower score group (7.7 % vs. 4.0 %, HR 2.03, 95% CI: 1.14-3.58, p=0.014) (Figure 2B). However, we did not see any significant difference in all-cause mortality between both groups (22.0 % vs. 17.9 %, HR 1.25, 95% CI: 0.94-1.65, p=0.127) (Figure 2C).

Prognostic impact of HFA-PEFF score

Two multivariate analyses demonstrated that an HFA-PEFF score ≥5 failed to predict CV mortality (aHR 1.37, 95% CI: 0.90-2.08, p=0.140) and time to first heart failure-related rehospitalisation (HR 1.49, 95% CI: 0.83-2.65, p=0.181). Atrial fibrillation was a strong independent predictor for both CV mortality (aHR 1.69, 95% CI: 1.14-3.26, p=0.011) and time to first heart failure-related rehospitalisation (aHR 2.84, 95% CI: 1.61-5.02, p<0.01). A history of the previous stroke was an independent predictor for CV mortality (aHR 1.93, 95 % CI: 1.14-3.26, p=0.015). The results of the univariate and multivariate analyses are shown in Tables 2-3.

**Table 2:** Univariate and multivariate analyses for cardiovascular mortality AF: atrial fibrillation; BMI: body mass index; DM: diabetes mellitus; CKD: chronic kidney disease; CAD: coronary artery disease; COPD: chronic obstructive pulmonary disease; MI: myocardial infarction; PCI: percutaneous coronary intervention; CABG, coronary artery bypass graft.

	Univariate analysis			Multivariate analysis		
	HR	95% CI	P-value	HR	95 % CI	P-value
Age	1.05	(1.01–1.09)	0.009	1.03	(0.99–1.08)	0.128
BMI	0.98	(0.94–1.02)	0.331			
DM	1.14	(0.71–1.84)	0.570			
Hypertension	1.04	(0.55–1.95)	0.892			
CKD	1.84	(1.18–2.84)	0.006	1.48	(1.93–2.38)	0.102
CAD	1.27	(0.82–1.94)	0.282			
Previous MI	1.01	(0.68–1.48)	0.955			
CABG	1.19	(0.86–1.65)	0.291			
Previous stroke	1.94	(1.53–3.29)	0.013	1.93	(1.14–3.26)	0.015
AF	1.92	(1.28–2.87)	0.001	1.69	(1.13–2.54)	0.011
COPD	1.49	(0.87–2.56)	0.142			
HFA-PEFF ≥ 5	1.58	(1.05–2.38)	0.029	1.37	(0.90–2.08)	0.140

**Table 3:** Univariate and multivariate analyses for heart failure-related rehospitalization AF: atrial fibrillation; BMI: body mass index; DM: diabetes mellitus; CKD: chronic kidney disease; GFR: glomerular filtration rate; CAD, coronary artery disease; MI: myocardial infarction; PCI: percutaneous coronary intervention; CABG: coronary artery bypass graft; COPD: chronic obstructive pulmonary disease.

	Univariate analysis			Multivariate analysis		
	HR	95% CI	P-value	HR	95% CI	P-value
Age	1.09	(1.04–1.16)	0.001	1.06	(1.00–1.12)	0.050
DM	1.00	(0.53–1.87)	0.997			
Hypertension	3.29	(0.8–13.5)	0.098	2.83	(0.68–11.62)	0.150
CKD	3.45	(1.73–6.88)	<0.001	2.32	(1.12–4.82)	0.024
CAD	0.98	(0.56–1.72)	0.955			
Previous MI	0.49	(0.12–2.02)	0.326			
CABG	1.13	(0.74–1.73)	0.582			
Previous stroke	0.92	(0.61–1.42)	0.721			
AF	3.40	(1.94–5.98)	<0.001	2.84	(1.61–5.02)	<0.001
COPD	1.04	(0.68–1.59)	0.859			
HFA-PEFF ≥ 5	2.03	(1.14–3.58)	0.016	1.49	(0.83–2.65)	0.181

## Discussion

We assessed the prognostic impact of the HFA-PEFF score in a large prospective registry with a longer follow-up by including 1,022 patients who have undergone TAVI for severe aortic stenosis. The main findings of our study are: (i) patients with an HFA-PEFF score ≥5 were older, had a higher prevalence of comorbidities (atrial fibrillation and chronic kidney disease), and had a higher estimated surgical risk. (ii) At two years of assessment, an HFA-PEFF score ≥5 was not an independent predictor of first heart failure-related rehospitalisation and CV mortality after TAVI, although the cumulative risks of both events were significantly higher in patients with an HFA-PEFF score ≥5 than in the lower score group. (iii) Atrial fibrillation was a strong independent predictor of CV mortality and first heart failure-related rehospitalisation.

More than half of heart failure patients undergoing TAVI are diagnosed with HFpEF and experience similar rehospitalisation and mortality rates when compared with other subsets of heart failure [10]. Yet, they could be misdiagnosed, and their management might be delayed [11-13]. We investigated the characteristics and long-term outcomes of this group of patients when identified by the HFA-PEFF score.

HFpEF is associated with co-morbidities that contribute to the progress of diastolic heart failures such as atrial fibrillation and chronic kidney disease [14,15]. We found that these co-morbidities were associated with a high HFA-PEFF score and some even strongly predicted adverse events, as in the case of atrial fibrillation or the history of a previous stroke. However, extending the use of this score for the prediction of HFpEF in patients with aortic valve diseases as in typical HFpEF patients might not be ideal. The phenotype similarity of patients with valvular heart diseases might mimic a typical HFpEF presentation but warrants a different approach to estimate their risk. Additionally, further remodelling of the heart chambers and haemodynamics after TAVI implantation in these patients might affect their pre-procedurally calculated score and allocate them to a different category afterward, which weakens the robustness and accountability of the HFA-PEFF score in patients with severe aortic stenosis.

In a recent observational study, a high HFA-PEFF score of a ≥5 was an independent predictor of all-cause mortality over 12 months. However, echocardiography parameters in the study were obtained after TAVI and long-term outcomes beyond 12 months were not assessed [6].

Another score-based algorithm, the H2PEF score, was tested for its generalisability and validity in HFpEF patients [16,17]. It proved its value as an independent predictor of adverse outcomes [18]. Contrary to our results regarding the HFA-PEFF score, a higher H2PEF score acted as an independent predictor of adverse cardiovascular and heart failure outcomes among patients with preserved EF undergoing TAVI [19]. Although both scores, the H2PEF score and the HFA-PEFF score, rely on clinical and echocardiographic parameters, a discrepancy between their results and how patients are assigned by their results to different approaches was reported [20,21]. Also, when both scores were applied in a prospective cohort to confirm the diagnosis of HFpEF, additional testing and invasive measures were required in up to half of the patients when the HFA-PEFF score was used and in up to one-third of the patients in the case of the H2PEF score [20]. The lack of broad applicability of these scores raises concerns about their usefulness in daily clinical practice. This could explain why in the latest European Society guidelines for the diagnosis and treatment of acute and chronic heart failure, a simplified pragmatic approach was endorsed for use instead of these scores [22].

Limitations

All echocardiographic interpretations in the study were obtained from our local hospital findings and were not controlled by a core laboratory. A modest disagreement on some variable values that are used in the score could lead to a change in our findings. Another concern that we must address is that we did not study the impact of the functional and final aetiological assessments (the last two steps of the HFA-PEFF algorithm). These steps could help in further assessing the prognostic value of the score.

Our data come from a prospective cohort, with the similarity in baseline characteristics of patients and the unified intervention with a long follow-up that enabled capturing larger numbers of events, all of which are points of strength in our study. 

Implication and future perspective

The use of prespecified risk scores such as the HFA-PEFF score could overcome diagnostic difficulties in HFpEF. However, this might not be applicable in patients with concomitant aortic valve diseases. Further research and careful examination of specific predictors of adverse events related to these patients are needed. A tailored approach using these predictors, combined with some established variables from available scores, is important to develop a validated score that can be applied to patients presenting with aortic valve diseases.

## Conclusions

We observed a limited value of the HFA-PEFF score in predicting long-term adverse events in patients undergoing TAVI for severe aortic stenosis with preserved EF. The higher calculated HFA-PEFF score for patients from our studied population failed to predict their all-cause mortality, CV mortality, and first heart failure-related rehospitalization.

Complementing the HFA-PEFF algorithm with clinical variables specific to patients with severe aortic stenosis may help to use it for daily clinical practise as a validated risk prediction tool.
